# The mental condition of Polish adolescents during the COVID-19 pandemic and war in Ukraine

**DOI:** 10.3389/fpubh.2023.1257384

**Published:** 2023-10-16

**Authors:** Małgorzata Wójtowicz-Szefler, Izabela Grzankowska, Monika Deja

**Affiliations:** Department of Psychology, Kazimierz Wielki University, Bydgoszcz, Poland

**Keywords:** COVID-19 pandemic, war in Ukraine, mental condition, adolescents, Polish population

## Abstract

Recently, the experience of the COVID-19 global pandemic has significantly affected the mental condition of entire societies by increasing anxiety and stress resulting from its sudden and completely unexpected nature. In Poland, apart from the pandemic, there is an ongoing threat of an armed conflict just across the border, which can constitute direct and indirect threats to physical and mental health. Each of these situations is unusual and difficult. It is also in sharp contrast to the developmental needs of children and adolescents. It especially violates the principal need of this developmental period, which is to grow up in a predictable as well as physically and emotionally safe environment. The purpose of the conducted research was to assess the psychological condition of Polish adolescents, whose social situation is difficult, in order to take appropriate preventive measures based on this assessment. The study was conducted using the “Who are You?” Scale of Transparency Anxiety and the Revised Child Anxiety and Depression Scale (RCADS), as well as a researcher-made survey containing questions about well-being in relation to the pandemic, distance learning and the ongoing war in a neighbouring country. Approval was obtained from the Bioethics Committee to conduct the projected research. The study included 945 adolescents aged 11 to 15 (M = 13.10; SD = 1.11) making a representative sample of adolescents from 14 regions in Poland. The results of the research and analyses show that the adolescents under study have a medium level of intensity of neuroticism, with the highest levels occurring in adolescents aged 14. Moreover, the adolescents manifest relatively lower symptoms of anxiety and depression as well as concern about the pandemic and the war in Ukraine than expected.

## 1. Introduction

The recent stressful experience of the COVID-19 global pandemic has significantly affected the psychological condition of entire societies by increasing anxiety and stress resulting from its sudden and completely unexpected nature (1, 2). In Poland, as in all Europe, social isolation and distance learning during the pandemic disrupted the lives of adolescents (3).

As stipulated in the guidelines of the Ministry of Health, the state of epidemic in Poland was in force between 15 March 2020 and 15 May 2022. Starting on 11 March 2020, the Polish government introduced distance learning for educational institutions (this did not, however, apply to special needs schools, educational and sociotherapy centres, centres for psychological guidance and educational counselling and schools at juvenile correctional centres and penal institutions). Starting on 4 May 2020, nurseries and pre-schools returned to in-person education, while primary and secondary schools continued distance learning until the end of June 2020, i.e., until the end of the school year. In the meantime, a variety of restrictions was introduced in Poland, such as restrictions on the freedom of movement, the obligation to wear masks, a ban on gatherings of more than two people, and a regulation stipulating that persons under 18 could only be in public spaces with an adult guardian. Shopping centres, service outlets and points-of-sale, entertainment venues such as cinemas, bars and discos were closed. All educational institutions started the school year 2020/2021 in the in-person mode, yet as early as on 24 October 2020, Grades 4 to 8 and secondary schools started distance learning, and on 4 November were joined by Grades 1–3. On 18 January 2021, in-person education was reinstated in Grades 1–3 and special needs schools. All educational institutions returned to full-time in-person education on 19 April 2021. However, even after this time, many classes or entire schools operated periodically in the distance learning mode as the identification of COVID-19 patients among students or teachers resulted in the quarantine of all other persons who had contact with them, and thus the transition to distance learning (4). This situation lasted until the end of the 2021/2022 school year. At times, some restrictions were either eased or tightened (due to the increasing number of cases), and this frequently occurred overnight. In spring 2021, the Polish government decided to massively ease restrictions.

For the Poles the war in the neighbouring country, which broke out in February 2022, turned out to be an additional factor that potentially increased the sense of social anxiety (5). The research conducted in various war-torn regions of the world demonstrated that children or adolescents who witnessed the death of or injuries inflicted upon their close relatives, or their torture or detention, were under fire or experienced physical violence themselves, as a consequence of these events developed disorders in their general functioning and experienced negative changes in their mental health. Admittedly, most children indirectly affected by war do not have to experience drastic consequences of the war: separation from their families or loved ones, abandoning their homes and hometowns, or losing their friends. They do, however, experience social threat through participation at a collective level, if only by hearing news of ongoing hostilities (6), or also through contacts with refugees who are victims of war. The experience of war, even that behind a neighbouring border, can result in perceiving a sense of danger and vulnerability in the face of the event, which is the essence of experiencing trauma (7). This implies that the mental health risks to children and young people arising from war may be indirect.

Both situations therefore contributed to the development of psychological stress in the Polish society due to their objectively burdensome and unusual nature. For this reason, it seems reasonable to answer the question whether the situation-related higher sense of threat caused by both the pandemic and the war in Ukraine had adverse consequences for the mental health of Polish adolescents in the form of increased symptoms of anxiety and depressive disorders, and whether these symptoms are linked to neuroticism as a personality trait of Polish adolescents.

The COVID-19 pandemic disrupted daily life not only through the physical health concerns raised, but it also resulted in numerous restrictions. Extreme precautions, such as mandatory social distancing, strict hygiene measures including wearing face masks as well as restrictions on the freedom of movement, have been taken due to the public health emergency. Many people in different parts of the world were able to cope well with this crisis experience and to keep their mental balance (8, 9). Some researchers have even indicated the possibility of emerging from stressful events with increased resilience (2, 9). Many people have been experiencing mental health problems as a result of the pandemic and its effects. Stress, anxiety and depression were most frequently recorded among the general population. However, among different social groups, it is the adolescents who may be most vulnerable to the biopsychosocial stressors generated by the pandemic (10). The occurrence of anxiety and depressive symptoms is among the most frequently observed mental disorders resulting from the COVID-19 pandemic on every continents of the world (11).

In addition to the pandemic, there is an ongoing threat resulting from the armed conflict directly across the Polish border. The experience of mass immigration is a confrontation with the realities of war and makes us realise the existing threat. A sudden influx of refugees and their long-term presence may be the cause of significant and previously unexpected changes in everyday life. The entirety of this experience fulfils the criteria of a difficult or even crisis situation due to the awareness of the threat, difficulties in achieving everyday goals, and information overload related to the influx of negative news (12). It leads to exposure to direct and indirect risks to physical and mental health, which is particularly debilitating for children and adolescents, as it limits their developmental opportunities. These experiences are in sharp contrast to developmental needs, especially the central need for all children and adolescents to grow up in a predictable as well as physically and emotionally safe environment (6). The experience of war just across the border may result in the sense of danger and vulnerability, which is the essence of experiencing trauma (7, 13). In view of the previous experience of the COVID pandemic, which significantly strained the coping capabilities of children, adolescents and whole families, the ongoing war generates an additional potential sense of threat that young people and their caregivers across Europe have to cope with. Children and young people in Poland experience it particularly acutely, not only because of the geographical proximity of the war and military operations, but also because of direct contacts with war victims. In 2022 year 11.8 million Ukrainian refugees, mainly women and children, arrived in Poland, of whom more than 10 million returned to Ukraine at the time of writing this paper. However, this means that during the year an unusually large group of children with traumatic experiences stayed in Poland and was supported not only by adults: many of their Polish peers, whether in educational institutions or during informal contacts within the family or the local community, have experienced accompanying a colleague from Ukraine in a traumatic experience or have heard about such events from relatives or the media. This situation can be also traumatising as it involves participating in the experience of extreme danger felt by someone else (14). The field literature sees this type of experience as a risk of vicarious trauma (7) or secondary post-traumatic stress disorder (15), as traumatic events affect the mental health of those accompanying the suffering person, thus causing an increased risk of adaptation difficulties in the form of potential emotional disorders in various forms.

Uncertainty resulting from real threats to safety, health and life is a particularly difficult experience for children and adolescents because their ability to cope with stress is incomplete, which is due to the early developmental stage (16–18). Children and adolescents are more vulnerable to environmental threats and situational stress because their cognitive performance and emotional maturity is lower than that of adults. This may result in difficulties in understanding stressful situations and in adopting a broader perspective alternative to the sense of threat, and may lead to using fewer coping strategies to deal with sudden changes arising from difficult events and situations (19).

Vulnerability to the development of anxiety and other internalising and externalising problems in adolescence can be explained by biochemical imbalances in the CNS during the period of intense developmental change as well as by higher levels of emotional reactivity and neuroticism (20–22). There are studies indicating a link between neuroticism and internalising psychopathology (i.e., distress, anhedonia, anxiety, fears of specific stimuli, social anxiety, and depression) (23). Neuroticism primarily reflects individual differences in the tendency to experience negative emotions and is a strong risk factor for the development of anxiety and depressive symptoms. Research by Williams et al. (23) suggests that neuroticism is a robust predictor of an adverse developmental trajectory for generalised stress leading to the development of psychiatric disorders. This was also confirmed by previous research conducted during the COVID-19 pandemic, which found that individuals who exhibit higher neuroticism experience more mental health problems (24). The findings of a study of Indian adolescents demonstrated that there is a strong positive correlation between neurotic personality trait, mental stress and perceived COVID-19-related stress. Thus adolescents with higher neuroticism are more vulnerable to mental stress during the COVID-19 pandemic. One may therefore conclude that neuroticism underlies vulnerability to stress in a proportion of the population (8), which accounted for a prediction of COVID-19-related stress of 73.4 per cent in the population of Indian adolescents with neurotic personality (25). In psychology, when looking for determinants of affective states, the sources of anxiety are often traced back to personality-related predispositions of the individual. Eysenck’s (26, 27) three-factor model of personality distinguishes three personality dimensions: neuroticism, extraversion and psychoticism. Anxiety is one of the main symptoms of high levels of neuroticism. Neurotics have a greater tendency to excessively experience negative emotions and to dwell on stress, as well as to overly react with anxiety (26). This constant tendency to experience negative emotions such as fear or anxiety, which includes a strong component of negative thinking and a tendency to react with negative emotions, is defined in C.D. Spielberger’s theory as identical with anxiety (a trait), as opposed to anxiety (a state) (28). Anxiety (a trait) is an established disposition to perceive various life situations as strongly threatening. It is shaped into a predisposition as a result of life experiences in the early years of development. It results in significant limitations in coping with challenges, which easily become stressful when perceived by a person with high anxiety levels. In this sense, a high level of neuroticism conditions a constant disposition to experience increasingly higher anxiety in difficult circumstances, and thus the situation of neurotic people who struggle with rough experiences can be particularly stressful, as the personal disposition to respond with anxiety will create a high risk in an anxiety-provoking situation. It is also worth remembering that not every anxiety reaction is a disorder, as some of them are relevant to the situation and subside with the disappearance of a threatening stimulus, which particularly concerns personalities with non-neurotic traits (28). The importance of protective factors that affect the intensity of subjectively experienced stress is also known. Due to individual dispositions, the same event may be burdensome and traumatising for one person, while for an individual with a different mental construct it will be merely a difficult challenge (29). Among the significant protective factors that affect the psychological condition of children and adolescents in a difficult situation one can identify coping strategies for dealing with stress. The array of the coping mechanisms changes with age to produce in adolescence a complex competence to regulate emotions as a result of an increase in metacognitive and self-regulatory abilities (17). Although the ability to use effective coping strategies varies during adolescence, yet due to cognitive competence it provides an important foundation for maintaining balance. Adolescents’ coping efficacy is also conditioned by temperamental traits, the resultant resilience, impulse control, and adaptability which helps to find adaptive solutions and protects against the dominance of negative emotions (17, 30).

Previous research conducted in Poland shows that about 10% of young people had mental problems (31, 32). A survey conducted in Poland in June 2021 (i.e., at the end of the school year during the Covid-19 pandemic online learning experience) found that approximately 15 per cent of pupils in Poland need significant intervention related to their mental functioning due to poor well-being (33). At the same time, some reports have implied that adolescents have psychological well-being after all, as they prefer active strategies to cope with threatening situations and they spend most of their time online (30). Other reports suggest that young people used their time mainly for distance learning, communication with peers and entertainment. The pandemic-generated precedent of pursuing new habits such as distance learning and online education (34) also meant that, despite the resourcefulness of young people, the pandemic was associated with increased uncertainty as to a previously unfamiliar way of life, although in the adolescent population this phenomenon was not that much intensified (35).

Obviously, the impact of the pandemic on the mental health of children and adolescents does not always manifest itself so dramatically. In practice, it can occur in a wide variety of forms, starting from a persistent lower mood over a long period of time, through more or less serious problems in relationships with others or in learning, to the onset (or exacerbation) of symptoms of generalised anxiety.

In China, where the pandemic started, approximately 35% of the general population experienced mild to severe distress due to COVID-19 (10, 36). Analyses conducted by Chinese researchers using the CPDI (COVID-19 Peritraumatic Distress Index) showed that young people, under 18 years of age, had the lowest scores in this study [mild distress; mean (SD) = 14.83 (13.41)]. The low level of distress in adolescents was accounted for by the relatively low incidence rate in this age group and limited exposure to the pandemic due to home quarantine. In contrast, an analysis of papers with significant contributions to the health of adolescents and young adults in the United States (The Distinguished Dozen: 2022) demonstrated an essential impact of the pandemic on the mental health and well-being of young people. According to Hertz et al. (37), who conducted a cross-sectional survey of COVID-19-related experiences among adolescents aged 13–19 from October to November 2020, American students in Grades 7–12 reported poorer mental health. Moreover, Runkle et al. (38), who studied suicidal behaviours in response to the COVID-19 pandemic among adolescents in the USA, identified four distinct crisis profiles: (a) depression/isolation/self-harm (10.4%), (b) interpersonal stress/depressed mood and anxiety (18.2%), (c) suicidal thoughts/depression (19.0%), and (d) adjustment/stress (52.4%). Following the study findings, they concluded that during the pandemic, in the subtypes of depression/isolation/self-harm and suicidal thoughts/depression there was an increase in suicidal thoughts and active rescue efforts (38).

Similar studies on the mental health of adolescents have been conducted in Europe. Data from a report by the OECD and the European Commission (39) show that the number of young people with depressive symptoms more than doubled in most EU countries. Young people were more likely than those in older age groups to view their mental condition as worse. Above all, there was an increase in the frequency of reported suicidal thoughts, although these were not associated with an increase in youth suicide rates. The incidence rate of anxiety in young people was also higher than before the pandemic. Gender-based disparities in the psychological condition of children and adolescents also increased. This means that young women were more likely to have symptoms of depression and anxiety than young men.

Taylor’s (40) research suggests that the presence or absence of anxiety is an important component of behaviour during an epidemic or pandemic. Taylor et al. (40) concluded that during the COVID-19 pandemic, research participants most commonly exhibited five interrelated anxiety reactions, which were collectively termed as the COVID stress syndrome. These included: (1) fear of the danger of contracting SARSCoV2, (2) concern about the economic consequences of the COVID-19 pandemic, (3) xenophobic concerns about foreigners transmitting the Coronavirus, (4) traumatic stress symptoms related to direct or indirect experience of the disease (nightmares, intrusive thoughts or images related to COVID-19), and (5) compulsive checking and support-seeking related to the COVID-19 pandemic.

These reports have also been confirmed in other studies conducted among, e.g., young people in France and Spain (41, 42). According to research conducted in France, the number of adolescents suffering from anxiety increased significantly, and out of those who experienced anxiety during the pandemic, a significant proportion also had moderate or severe depression or experienced severe stress. In Spain, by contrast, 50.43% of adolescent respondents experienced the effects of the pandemic, of whom 21.34% experienced moderate to severe anxiety, 28.14% stress and as many as 34.19% depression.

The aim of this study was to determine whether Polish adolescents go through excessive experiencing of negative emotions and dwell on stressful events in a critical situation such as the COVID-19 pandemic and the war in Ukraine. The study set out to identify the relationship between neuroticism and the level of severity of anxiety and depressive symptoms, and to investigate whether changes associated with a persistent sense of threat might have adverse consequences in terms of the severity of symptoms of anxiety and depressive disorders in Polish adolescents. Moreover, a decision was taken to verify whether there are gender and age-related differences in the level of anxiety and depression severity, and whether there is an interplay between anxiety and depression severity. The research was conducted in order to propose evidence-based preventive interventions to young people.

## 2. Materials and methods

### 2.1. Research group

A total of 945 adolescents aged 11 to 15 (M = 13.10; SD = 1.11) participated in the study. The study was conducted on a representative sample of adolescents who came from 14 regions in Poland (out of 16 existing regions). The demographics of participants are presented in Table 1. The study was conducted in groups and took place in classrooms in May and June 2022. The questionnaires were prepared in electronic format. Young people taking part in the survey were given an individual identification code (ID) to access the online form. The questionnaire included instructions on how to proceed. The adolescents were accompanied during the study by persons prepared to give additional instructions (a school counsellor or psychologist). The student’s parents or legal guardians gave their consent to the student’s participation in the study. The adolescents gave their informed consent to participate in this study.

**Table 1:** Characteristics of study participants.

	N (*N* = 945)	%
Gender		
Girls	520	55.03%
Boys	425	44.97%
Age		
11	70	7.37%
12	232	24.42%
13	291	31.16%
14	243	25.58%
15	109	11.47%
Place of school attendance		
Village	325	34.39%
Small town up to 20.000 residents	199	21.06%
Big city over 20.000 residents	228	24.13%
Major city over 100.000 residents	193	20.42%
Mother’s education		
Primary	59	6.24%
Vocational	184	19.47%
Secondary	240	25.40%
Higher	462	48.89%

### 2.2. Research tools

The “Who are You”? Scale of Transparency Anxiety (WaY) by Elżbieta Skrzypek and Mieczysław Choynowski, adapted by Zwierzyńska and Matuszewski (28) was applied in the study. The scale is used to measure anxiety (a trait) as an indicator of neuroticism as two components: cognitive and emotional-physiological. This tool was standardised in a group of children from Grades 4–6 of primary school and Grades 1–3 of lower secondary school, which currently corresponds to the age group of children and adolescents from 11 to 16 years of age. The WaY scale includes 50 statements, to which the subject is asked to respond by selecting YES or NO answers. The Neuroticism Scale (N) consists of 40 statements, with maximum score of 40 points. The confidence interval of +/− 3 points in relation to the raw score distribution is also identified for this scale. This scale also includes a control score, namely the Scale of Lying (L) consisting of 9 statements, which indicates socially expected behaviours and provides information about the intensity of the respondent’s desire to seek social approval. The reliability of the test, measured by the Cronbach’s α coefficient and set by the authors is 0.86, while in the study group the Cronbach’s α is 0.89, indicating a high reliability of the tool.

The Revised Child Anxiety and Depression Scale RCADS by Chorpita et al., in its Polish adaptation by Ilona Skoczeń et al. (43) was applied in the study. Its objective is to assess the severity of symptoms of anxiety and depressive disorders in children. It allows to determine the anxiety and depression index (GLOBAL) as well as indices of Separation Anxiety Disorder (SAD), Generalised Anxiety Disorder (GAD), Panic Disorder (PD), Social Phobia (SOC), Obsessive-Compulsive Disorder (OCD) and Major Depression Disorder (MDD) based on the clinical criteria in the DSM-5 classification. The original English version of the tool is aimed at children and adolescents aged 3 to 17.5. The Polish version of the questionnaire was used on a sample of 501 children and adolescents aged 8 to 14 (43). The questionnaire contains 47 statements that need providing the information on the frequency of occurrence of a given symptom on the scale from “Never,” “Sometimes,” “Often” to “Always.” Interpretation of the results should be done with caution, as research on this tool has been still ongoing (43, 44). The reliability of the questionnaire, assessed by the authors in compliance with the Bagozzi’s formula, is 0.96. In this study, the reliability measured by Cronbach’s α coefficient is 0.99.

The researcher-made survey consisted of three questions that addressed reactions to events related to the pandemic and the war in Ukraine. Respondents assessed on a scale of 0 (no concern) to 10 (very strong concern) their level of concern about the pandemic and the war in Ukraine, as well as the level of stress experienced during distance learning. Each question could be scored from 0 to 10.

The following questions were asked:

Q1. How much anxiety did you feel about the COVID-19 pandemic?

Q2. How would you assess the stress you usually feel during distance learning?

Q3. How much anxiety did you feel about the war in Ukraine?

### 2.3. Statistical analysis

Statistical analysis was performed using the STATISTICA software. The following methods were used: Kruskal-Wallis ANOVA rank test, Student’s *t* test for significance of differences between groups, Pearson’s *r* correlation coefficient and Chi-square correlation coefficient and linear regression analysis.

## 3. Results

### 3.1. Descriptive statistics

Table 2 presents the descriptive statistics of the examined variables, derived from, e.g., Revised Children’s Anxiety and Depression Scale (RCADS) which allows to measure the anxiety and depression index (GLOBAL) and the indices of Separation Anxiety Disorder (SAD), Generalised Anxiety Disorder (GAD), Panic Disorder (PD), Social Phobia (SOC), Obsessive Compulsive Disorder (OCD) and Major Depression Disorder (MDD), the “Who are you?” Transparent Anxiety Scale (WaY), which measures neuroticism (N) and the desire to seek social approval (L) as well as answers to questions from the researcher-made survey.

**Table 2:** Descriptive statistics of the study variables.

Variables	M	SD	Min	Max	Skewness	Kurtosis
RCADS						
SOC	3.50	2.05	0.00	9.00	0.38	−0.08
PD	1.65	1.57	0.00	9.00	1.41	3.00
MDD	6.40	3.47	0.00	15.00	0.29	−0.47
SAD	2.08	2.03	0.00	9.00	1.09	0.89
GAD	2.26	1.97	0.00	9.00	0.93	0.67
OCD	2.34	2.08	0.00	9.00	0.93	0.47
GLOBAL	39.89	26.42	0.00	141.00	0.94	1.07
WaY						
Lying	5.26	1.59	0.00	9.00	−0.59	0.93
Neuroticism	16.78	8.55	0.00	40.00	0.18	−0.55
Survey						
Q1	3.52	2.80	0.00	10.00	0.45	−0.74
Q2	2.50	2.70	0.00	10.00	1.07	0.30
Q3	4.75	3.06	0.00	10.00	0.03	−1.06

SOC, social phobia; PD, panic disorder; MDD, major depressive disorder; SAD, separation anxiety; GAD, generalised anxiety; OCD, obsessive-compulsive disorder; GLOBAL, the total anxiety and depression index; L, scale of lying; N, neuroticism scale; Q1, anxiety about the pandemic. Q2, distance learning. Q3, the war in Ukraine.

### 3.2. Level of neuroticism and social approval

Firstly, it was tested whether the levels of neuroticism and desire to seek social approval of the adolescents studied were gender-based (Table 3) and age-based (Table 4). The Student’s *t* test for independent data was applied. As shown in Table 3 gender does not differentiate the level of desire to seek social approval in the adolescents under study, as expressed on the Scale of Lying, as presenting oneself in a better light (t = −0.579; df = 943; *p* = 0.563). Girls, however, manifest significantly higher levels of neuroticism than boys (t = −9.706; df = 943; *p* < 0.001), and this effect is moderate (d = 0.64). The anxiety level of the surveyed adolescents can be described as average (boys – sten score of 5–6, girls – sten score of 6). It was then tested whether age is a differentiating factor for anxiety and need for social approval. A non-parametric Kruskal-Wallis ANOVA rank test was used due to the large disparity in the volume of the subgroups (Table 4). The analyses showed that age did not affect the level of need for social approval [H(4, *N* = 945) = 3.68; *p* = 0.452] and the level of neuroticism [H(4, *N* = 945) = 5.84; *p* = 0.211] among the participants.

**Table 3:** Differences between boys and girls in the desire to seek social approval (L) and neuroticism (N).

	Boys		Girls		*t*(943)	*p*-value	Cohen’s *d*
	M	SD	M	SD			
WaY							
Lying	5.23	1.70	5.29	1.49	−0.579	0.563	-
Neuroticism	13.93	8.06	19.11	8.23	−9.706	<0.001	0.64

**Table 4:** Differences in levels of social approval (L) and neuroticism (N) by age.

	11		12		13		14		15		*H* (4, *N* = 945)	*p*-value
	M	SD	M	SD	M	SD	M	SD	M	SD		
WaY												
Lying	5.04	1.93	5.23	1.52	5.32	1.54	5.37	1.53	5.06	1.76	3.68	0.452
Neuroticism	15.19	9.88	16.87	8.09	16.71	8.52	17.58	8.48	16.00	8.76	5.84	0.211

The age-related (Table 5) and gender-related (Table 6) distribution of the level of neuroticism of the adolescents represented by low (sten score of 1–4), medium (sten score of 5–6) and high (sten score of 7–10) scores was also investigated. The correlations obtained, as measured by the Chi-square coefficient, confirmed previous findings on the significance of gender and age for anxiety levels. Moreover, they allowed to illustrate the percentage distribution of results (low, medium or high scores). The abundance tables presented show that the highest number of subjects reached high values, i.e., above sten score of (40.42% of subjects). This means that a significant proportion of the adolescents surveyed demonstrate an excessive tendency to experience stress and to dwell on difficult situations. The highest percentage of high scores is observed among adolescents aged 14 (43.62%), and the lowest among 11-year-olds (33.33%). In addition, the youngest adolescents, i.e., 11-year-olds, most frequently (42.03%) revealed a low level of neuroticism. High scores were also more frequent among girls (44.04%) compared to boys (36.00%).

**Table 5:** Severity of neuroticism vs. age of respondents.

WaY N						
	11	12	13	14	15	N
Low	29 (42.03%)	59 (25.65%)	75 (25.42%)	60 (24.69%)	31 (28.70%)	254
Average	17 (24.64%)	78 (33.91%)	103 (34.92%)	77 (31.69%)	34 (31.48%)	309
High	23 (33.33%)	93 (40.44%)	117 (39.66%)	105 (43.62%)	43 (39.82%)	382
Total	69	230	295	243	108	945

Chi^2^ = 10.39; df = 8; *p* = 0.239.

**Table 6:** Severity of neuroticism vs. gender of respondents.

WaY N			
	Boys	Girls	Both
Low	142 (33.41%)	112 (21.54%)	254
Average	130 (30.59%)	179 (34.42%)	309
High	153 (36.00%)	229 (44.04%)	382
Total	425	520	945

Chi^2^ = 17.06; df = 2; *p* < 0.001.

### 3.3. Level of anxiety and depression

The analyses aimed at whether there was a significant difference between boys and girls in terms of anxiety and depression as measured by the RCADS questionnaire. The Student’s *t* test for independent samples was applied. The results are presented in Table 7. The girls under study demonstrate higher anxiety and depression in all areas investigated such as social phobia (t = −7.963; df = 943; *p* < 0.001), panic disorder (t = −5.100; df = 943; *p* < 0.001), major depression disorder (t = −9.463; df = 943; *p* < 0.001), separation anxiety disorder (t = −6.905; df = 943; *p* < 0.001), generalised anxiety (t = −4.568; df = 943; *p* < 0.001), obsessive-compulsive disorder (t = −6.178; df = 943; *p* < 0.001), and in the total score (t = −8.328; df = 943; *p* < 0.001). These effects are weak to moderate.

**Table 7:** Differences between boys and girls in the levels of anxiety and depression.

	Boys		Girls		*t*(943)	*p*-value	Cohen’s *d*
	M	SD	M	SD			
RCADS							
SOC	2.93	2.00	3.97	1.98	−7.963	<0.001	0.52
PD	1.37	1.57	1.89	1.54	−5.100	<0.001	0.33
MDD	5.27	3.30	7.33	3.34	−9.463	<0.001	0.62
SAD	1.59	1.84	2.49	2.08	−6.905	<0.001	0.46
GAD	1.94	1.98	2.52	1.92	−4.568	<0.001	0.30
OCD	1.89	1.95	2.71	2.11	−6.178	<0.001	0.40
GLOBAL	32.25	24.87	46.14	26.02	−8.328	<0.001	0.55

It was also verified whether the age of the subjects affects the results obtained from the RCADS questionnaire (Table 8). The age of the adolescents had an impact only on the level of separation anxiety [H(4, *N* = 945) = 11.90; *p* = 0.018]. Post-hoc tests showed that 11-year-olds had significantly lower level of separation anxiety compared to 14- and 15-year-olds.

**Table 8:** Severity of anxiety and depression vs. age of respondents.

	11		12		13		14		15		*H* (4, *N* = 945)	*p*-value
	M	SD	M	SD	M	SD	M	SD	M	SD		
RCADS												
SOC	2.91	2.01	3.37	1.96	3.49	2.04	3.70	2.09	3.73	2.18	8.30	0.081
PD	1.67	1.75	1.66	1.53	1.56	1.51	1.66	1.51	1.89	1.86	2.11	0.715
MDD	5.54	3.68	6.53	3.42	6.64	3.43	6.43	3.47	5.95	3.51	7.77	0.100
SAD	1.57	1.99	1.90	1.88	2.11	2.03	2.28	2.08	2.30	2.17	11.90	0.018
GAD	2.09	2.20	2.17	1.88	2.24	1.91	2.33	1.90	2.48	2.28	2.90	0.575
OCD	2.03	2.16	2.42	2.04	2.38	2.07	2.28	2.01	2.39	2.30	3.75	0.441
GLOBAL	33.30	27.23	39.89	25.09	39.93	25.92	41.28	26.54	40.91	29.44	6.69	0.153

### 3.4. Concerns over the pandemic and war

The aim was to investigate whether there was a significant difference between boys and girls in terms of perceived anxiety related to the COVID-19 pandemic, distance learning and the war in Ukraine. Student’s *t* test for independent samples was used. The results are presented in Table 9. Girls were significantly more concerned than boys about the situation related to the COVID-19 pandemic (t = −5.180; df = 896; *p* < 0.001) and the war in Ukraine (t = −7.939; df = 896; *p* < 0.001), and significantly more stressed about distance learning (t = −6.815; df = 896; *p* < 0.001). These effects were weak to moderate.

**Table 9:** Differences between boys and girls in terms of concern about the world’s social situation.

	Boys		Girls		*t*(943)	*p*-value	Cohen’s *d*
	M	SD	M	SD			
Survey							
Q1	2.99	2.87	3.95	2.67	−5.180	<0.001	0.35
Q2	1.73	2.44	3.12	2.74	−7.939	<0.001	0.54
Q3	3.99	3.15	5.36	2.84	−6.815	<0.001	0.46

The assumption whether the age of the subjects affected the level of anxiety was also investigated (Table 10). The age of the subjects did not influence the level of concern about the pandemic, distance learning and the war in Ukraine.

**Table 10:** Level of concern about the world’s social situation.

	11		12		13		14		15		H (4, *N* = 898)	*p*-value
	M	SD	M	SD	M	SD	M	SD	M	SD		
Survey												
Q1	3.39	2.69	3.51	2.74	3.76	2.82	3.32	2.80	3.35	2.98	3.97	0.410
Q2	2.39	2.82	2.46	2.44	2.40	2.60	2.75	2.94	2.35	2.82	2.34	0.674
Q3	4.44	3.22	4.63	2.98	5.01	3.00	4.67	3.11	4.61	3.16	3.01	0.556

### 3.5. Dependencies between the variables under study

Finally, the level of neuroticism was measured to test whether it correlates with the symptom levels of anxiety and depression in the adolescents under study (Table 11), as well as the level of concern about the pandemic (Table 12), distance learning and the war in Ukraine.

**Table 11:** Correlation between neuroticism and adolescents’ anxiety and depression levels by gender.

	WaY N	
	Boys	Girls
RCADS		
SOC	0.622^***^	0.552^***^
PD	0.488^***^	0.390^***^
MDD	0.603^***^	0.599^***^
SAD	0.531^***^	0.587^***^
GAD	0.524^***^	0.484^***^
OCD	0.604^***^	0.529^***^
GLOBAL	0.713^***^	0.691^***^

*** *p* < 0.001.

**Table 12:** Subjects’ gender-related correlations between neuroticism and concern about the world’s social situation.

	WaY N	
	Boys	Girls
Survey		
Q1	0.324^***^	0.096^*^
Q2	0.399^***^	0.298^***^
Q3	0.304^***^	0.108^*^

* *p* < 0.05;

*** *p* < 0.001.

Analyses were performed separately for boys and girls due to the previously noted gender-related differences in the levels of these variables. Pearson’s r correlation coefficient was applied. Neuroticism levels correlate positively with all dimensions of anxiety and depression in both boys and girls. These correlations range from moderate to very strong. The level of neuroticism correlates also positively with the level of concern about the pandemic, distance learning and the war in Ukraine. These correlations are moderate in boys and weak in girls.

Moreover, it was observed that in boys there is a weak positive correlation between age and social phobia (r = 0.134; *p* < 0.01), separation anxiety (r = 0.155; *p* < 0.01), generalised anxiety (r = 0.101; *p* < 0.05) and the total anxiety and depression index (r = 0.101; *p* < 0.05), i.e., the older the boys are, the more anxious they are and the more prone to depressive reactions. In girls, there is a weak positive correlation between age and social phobia (r = 0.113; *p* < 0.05) and separation anxiety (r = 0.095; *p* < 0.05), i.e., the older the girls are, the more likely they are to be fearful of negative social evaluation and slightly more anxious about separation from their caregiver (Table 13).

**Table 13:** Relationships between severity of anxiety and depression and age of adolescents surveyed.

	Age	
	Boys	Girls
RCADS		
SOC	0.134^**^	0.113^*^
PD	0.089	0.008
MDD	−0.006	0.054
SAD	0.155^**^	0.095^*^
GAD	0.101^*^	0.036
OCD	0.030	0.026
GLOBAL	0.101^*^	0.066
WaY		
Lying	0.048	−0.024
Neuroticism	0.067	0.037
Survey		
Q1	−0.018	0.002
Q2	0.041	0.046
Q3	0.015	0.036

* *p* < 0.05;

** *p* < 0.01.

### 3.6. Neuroticism as a predictor of anxiety and depression

The linear regression analysis was applied to verify whether the level of neuroticism of the young people under study is a predictor of the general severity of anxiety and depression, which is reflected in the overall score in the RCADS questionnaire. The data are presented in Table 14. It turned out that neuroticism is a significant predictor of the general severity of anxiety and depression in the studied youth (t = 31.67; df = 943; *p* < 0.001) and accounts for 51.5% of variance of this outcome. It is also a predictor of the severity of all individual types of anxiety and depressive disorders, as measured by the RCADS scale (SOC - β = 0.61, t = 23.84, df = 939, *p* < 0.001, R^2^ = 0.21; PD - β = 0.46, t = 15.76, df = 939, *p* < 0.001, R^2^ = 0.38; MDD - β = 0.64, t = 25.26, df = 939, *p* < 0.001, R^2^ = 0.40; SAD - β = 0.59, t = 22.40, df = 939, *p* < 0.001, R^2^ = 0.35; GAD - β = 0.52, t = 18.54, df = 939, *p* < 0.001, R^2^ = 0.27; OCD - β = 0.58, t = 22.03, df = 939, *p* < 0.001, R^2^ = 0.34). The study results indicate the importance of the personality trait – neuroticism - for the occurrence of symptoms of emotional disorders in young people. Concurrently, this outcome suggests that a lower intensity of neuroticism may be, in a difficult situation, a protective factor for the emergence of anxiety and depression symptoms in the group of adolescents.

**Table 14:** Regression analysis of the variable sum of anxiety and depression.

Predictor	*b* ^*^	*S.e. of b* ^*^	*b*	*S.e. of b*	t(943)	*p*
Constant			2.67	1.32	2.02	0.043
Neuroticism	0.72	0.02	2.22	0.07	31.67	<0.001^***^

*F*(1, 943) = 1003.0; *p* < 0.001; Std. error of estimation: 18.40; *R*^2^ = 0.515. *p < 0.05;

*** *p* < 0.001.

## 4. Discussion

This study examined the severity of anxiety symptoms, represented by the occurrence of various anxiety symptoms, symptoms of depressive disorders, severity of neuroticism, and attitudes towards social approval in adolescents aged 11 to 15. The study was carried out during a period of particular exposure of adolescents to traumatising social factors, i.e., in the spring of 2022 in Poland, just after the start of the war in Ukraine and 2 years after the onset of the COVID-19 pandemic. However, the level of severity of neuroticism as a trait was found to be moderate in the population of adolescents studied, and it was gender-related, i.e., it was higher in girls than in boys. However, the higher intensity of neuroticism in girls is a long-observed and repeatedly corroborated trend in research (34, 45, 46). Yet, it is interesting to note that in the study sample, the biggest proportion of respondents obtained high scores of neuroticism, i.e., above sten score of 7 (40.42% of the entire study sample), which shows that the significant part of the total number of the adolescents studied is vulnerable to stress and anxiety. The highest severity of neuroticism is observed among adolescents aged 14. Perhaps this group was most severely affected by both these difficult and unusual phenomena, i.e., the pandemic and the war, during their development due to personality susceptibility, which allows to predict the occurrence of anxiety and depressive disorders. It largely depends on to what extent those difficult situations - the pandemic and the war - disrupted the functioning of young people. An important condition for maintaining balance in a situational crisis overlapping with a difficult developmental stage is the very construct of a young person’s personality. It also clarifies how individuals react differently to a common experience (45, 47).

Nonetheless, the results of the study showed relatively lower rates of symptoms of the observed disorders than expected. It turned out that the adolescents surveyed felt moderately concerned. Similar results were reported in a multicultural community study of adolescents in Singapore, where it was found that, despite the pandemic, about 50 per cent of the adolescents under study presented an average level of resilience, rather than a lower one, as it was assumed. Lower levels of resilience were specifically associated with poorer coping skills and lower socio-economic status, which in Singapore is the case of the minority of young people of Chinese origin (48).

In contrast, the group of the youngest adolescents surveyed (aged 11) demonstrated the highest percentage of low severity of neuroticism. At the same time, the profile of other age groups was similar in this respect. Through this inconsistency in the frequency distribution a developmental difference was probably identified and it demonstrated lower vulnerability to anxiety among young persons in preadolescence. This is consistent with the developmental dynamics, which is associated with an increase in the tendency towards anxiety and emotional instability in adolescents after the age of 12 in many cultural areas, regardless of the part of the world (44). The age-related difference in anxiety severity also relates to the prevalence of symptoms of selected anxiety disorders, which are fewer in adolescents at 11 years of age compared to their older peers. In the study group, a significant difference concerns separation anxiety, which is higher in older adolescents. In clinical terms, this is an indicator of a reduced sense of security, which is lower in adolescents aged 14 and 15. This is perhaps the result of emotional imbalance increasing with age, therefore there is a developmentally justified search for security in the relationship with parents (45, 48). However, it cannot be excluded, either, that adolescents express their concern with tragic events and with this infrequent developmental burden. It may have greater significance and stronger effects on the emotional state of older adolescents who perceive psychology-related changes more strongly and make more conscious judgements regarding the resources and risks present in the environment in comparison to the adolescents at an earlier stage of adolescence who are more focused on the biological changes (48). This effect is gender-related because boys, the older they get, are generally more anxious and prone to depressive reactions. In girls, on the other hand, it is expressed as an increase in the fear of negative social evaluation and separation from their caregiver: the older the girls are, the more likely they are to experience such fears. The differences identified confirm the knowledge of gender preferences, which in girls are significantly more related to sensitivity to social evaluation and approval-seeking, as partially indicated by the results obtained (49). The fact that boys are more prone to depressive reactions, however, is somewhat surprising in view of previous reports that girls in adolescence are also more susceptible to depression due to biochemical and emotional determinants (50). In an earlier study of Polish adolescents, the prevalence of depressive symptoms in girls was statistically significantly higher than in boys, at 33.6 per cent and 18.2 per cent for girls and boys, respectively (51). However, research on the assessment of the risk of depression among adolescents during the COVID-19 pandemic (52) does not indicate a gender advantage in terms of depressive reactions, which may suggest that the boys’ population bears the consequences of the burdensome situations on a par with girls.

The research presented also showed that adolescent girls experienced significantly higher anxiety at the time of the survey than the adolescent boys. Thus, there are significant gender-related differences in the severity of anxiety symptoms among the subjects, as it is the girls who have significantly higher stress indicators than the boys in the study group. Previous extensive research on this developmental period shows that female gender is a risk factor for anxiety (53, 54), and adolescent girls report many times higher levels of stress than boys in this age group (55, 56). The results of the studies mentioned confirm this trend. The girls under study have more depressive symptoms and a higher overall score for anxiety and depressive disorder symptoms, although the effect is not strong. Similar results were obtained in a study of Croatian adolescents published by a team of researchers from Rijeka (57), which showed that the average anxiety level of Croatian adolescents was not high. At the same time, girls also reported more anxiety symptoms, i.e., they felt anxious to a greater extent than boys in the Croatian study group.

The degree of concern about the pandemic and the war in Ukraine was also investigated by directly asking the youth surveyed about these issues. It turned out, as in the case of anxiety and depression symptoms, that girls were significantly more anxious than the boys about the COVID-19 pandemic and felt the stress related to distance learning more strongly. Moreover, their anxiety about the war in Ukraine was higher in the study group, although the effect of the observed differences was not strong. Perhaps the expectations of higher levels of anxiety and depression symptoms in adolescents are due to the search for analogies as to the way adults react to stress. However, this is age-determined (17), which is related to competence and predisposition in the cognitive assessment of a stressor. In earlier studies in adults, the propensity to anticipate catastrophic events and one’s own coping ability in the secondary assessment of the stressor were predictors of anxiety, which was not confirmed in the adolescent group (58). At the same time, ruminations turned out to be a factor reinforcing adolescents’ susceptibility to respond with anxiety, but this aspect of functioning was not investigated in the study group. The effect obtained in measuring the severity of anxiety of adolescents from Croatia is partly accounted for by the result showing that the adolescents under study are more likely to choose adaptive cognitive emotion regulation strategies than other strategies which potentially confirms the developmentally increasing ability to cope with crisis situations in this age group (18, 19).

However, the conducted research confirmed at least a moderate, and in some cases strong, association of neuroticism as a trait with the occurrence of psychopathological symptoms. In the group of adolescents studied, symptoms of anxiety were observed, including social phobia, panic anxiety, separation anxiety, generalised anxiety and obsessive-compulsive disorder, as well as symptoms of depression in connection with adolescents’ neuroticism. It turned out, as expected, that the higher the neuroticism, the higher the prevalence of all the aforementioned manifestations of anxiety and depression, both in boys and girls. These results are compliant with initial reports on neuroticism and its anxiety-predisposing role, which was confirmed by Eysenck (26, 27). He states that the dominance of negative emotionality and the tendency of neurotics to overindulge in stress generates developmentally adverse consequences in the form of overreacting with anxiety. The result obtained is all the more worrying as about 40 per cent of the respondents are highly neurotic, which denotes a very high proportion of adolescents at a particular risk of constantly experiencing significant levels of anxiety in difficult situations. Alarming reports – provided by professionals observing the mental health crisis of young people – about the need to improve psychological counseling and psychiatric support after the pandemic seem justified (47). Not only treatment strategies, but perhaps mainly prevention of anxiety and depression are currently a challenge in Poland, also due to the ominous threat of war. It is also a challenge worldwide to prevent the effects of objective threats from being imprinted in the experiences and personalities of young people. Enhancing young people’s coping competences may therefore be a challenge worth undertaking so as to effectively protect them from psychopathological effects even in such extreme experiences as the pandemic and the resultant social constraints, as well as the threat of an armed conflict caused by the war in the neighbouring country.

## 5. Conclusion

The findings of this study show that the average level of severity of neuroticism as a trait in the population of adolescents surveyed, aged 11–15, was moderate, although a significant proportion of the adolescents under study were highly vulnerable to stress and anxiety. The highest intensity of neuroticism was observed among adolescents aged 14, while the highest percentage of low level of neuroticism was found in the youngest adolescents surveyed (aged 11). The level of neuroticism varied by gender, i.e., it was higher in girls than in boys.

Moreover, the adolescents under study had relatively lower rates of anxiety and depression symptoms and concern about the pandemic and the war in Ukraine than expected. Cross-gender differences in experiencing symptoms of anxiety and depression were observed. The girls surveyed had higher levels of anxiety and depressive symptoms, and reported more anxiety about the pandemic and war in Ukraine. It turned out, as in the case of anxiety and depressive symptoms, that girls were significantly more anxious than boys about the COVID-19 pandemic and felt the stress of distance learning more strongly. Their anxiety about the war in Ukraine was higher in the study group, although the effect of the observed differences was not strong. Finally, it should be stressed that the age-related difference in the severity of separation anxiety was identified, which was higher in older adolescents (i.e., those aged 14 and 15), especially in boys. The older the adolescents are, the more anxious they are in general and more prone to depressive reactions. In girls, on the other hand, this is expressed by an increase in the fear of negative social evaluation and separation from the caregiver: the older the girls are, the more likely they are to experience such fears. The differences identified confirm the existence of gender-related preferences, which in girls are significantly more related to sensitivity to social evaluation and approval-seeking, and this is partly demonstrated in the results (49).

## 6. Limitations

Among the limitations of the study design presented, it is worth mentioning that the survey was conducted in groups via an online form, thus the young people provided self-reports on the basis of their own understanding of the survey questions. Although the procedure took place with the researcher present, it seems probable that some respondents may not have used the opportunity to ask for clarifications, in case the description of the statement was not well-understood. A questionnaire method was used, therefore the results only include information about the conscious experiences of the respondents, while the description of potential subconscious mental processes related to the suppression of unpleasant emotional states was not available in the study (59). Another limitation is the lack of information from parents that would enable to compare the results obtained from the adolescents, and this could be a significant contribution on the informative level as it is practiced in standard assessment procedures for children and adolescents. Due to the nature of the study, the research involved adolescents from a standard group participating in regular school education. This also means that on a day-to-day basis the respondents sufficiently adaptively cope with the demands of everyday life, i.e., they have the resources to cope with stress (17). It seems reasonable to study the same variables in children and adolescents burdened with various anxiety disorders, i.e., to investigate whether high-risk groups experience an increase in symptoms in the event of higher situational stress. When examining the emotional impact of difficult situations in adolescents, it would be essential to monitor the use of coping strategies and to measure the level of resilience, as both variables in previous studies have been found to be important in shaping young people’s individual responses to situational stress (57, 59). Conducting longitudinal and experimental studies in the future that could be useful for investigating prolonged exposure to stress factors (60) – which undoubtedly include the ongoing war in this part of Europe – is worth considering.

## Data availability statement

The raw data supporting the conclusions of this article will be made available by the authors, without undue reservation.

## Ethics statement

The studies involving human participants were reviewed and approved by the Ethics Committee of the Faculty of Psychology at the Kazimierz Wielki University in Bydgoszcz, Poland (7/13.06.2023). Written informed consent to participate in this study was provided by the participants’ legal guardian/next of kin.

## Author contributions

MW-S: Conceptualization, Data curation, Formal analysis, Investigation, Methodology, Project administration, Writing – original draft, Writing – review & editing. IG: Conceptualization, Data curation, Formal analysis, Investigation, Methodology, Project administration, Writing – original draft, Writing – review & editing. MD: Conceptualization, Data curation, Formal Analysis, Investigation, Methodology, Project administration, Writing – original draft.
